# Spatial FAP Expression as Detected by ^68^ Ga-FAPI-46 Identifies Myofibroblasts Beyond the Infarct Scar After Reperfusion

**DOI:** 10.1007/s11307-025-01994-6

**Published:** 2025-03-03

**Authors:** Annika Hess, Alexandra Renko, Andreas Schäfer, Mira Jung, Daniela Fraccarollo, Jan D. Schmitto, Johanna Diekmann, Thomas Thum, Frank M. Bengel, Johann Bauersachs, James T. Thackeray, Jochen Tillmanns

**Affiliations:** 1 Department of Nuclear Medicine, Hannover Medical School, Hannover, Germany; 2 Department of Cardiology and Angiology, Hannover Medical School, Carl-Neuberg-Str. 1, 30625 Hannover, Germany; 3 Institute of Molecular and Translational Therapeutic Strategies, Hannover Medical School, Hannover, Germany; 4 Department of Cardiothoracic Surgery, Hannover Medical School, Hannover, Germany; 5 nextGENERATION Medical Scientist Program (Hannover Medical School), Hannover, Germany

**Keywords:** Fibroblast activation protein α, Ischemia/reperfusion, Area at risk, Cardiac myofibroblast, Perfusion SPECT, 68 Ga-FAPI-46 PET

## Abstract

**Purpose:**

Myocardial infarction (MI) triggers complex cellular responses essential for tissue repair and remodeling, including myofibroblast activation. Fibroblast activation protein alpha (FAP) identifies activated myofibroblasts post-MI, however its spatial distribution relative to the scar and area at risk (AAR) is unclear. Non-invasive FAP-imaging with PET radiotracer ^68^ Ga-FAPI-46 shows uptake beyond the infarct scar. We therefore aimed to characterize FAP expression in the AAR using a myocardial ischemia–reperfusion (MI/R) model in mice.

**Procedures:**

We induced MI/R in male C57BL/6N mice. The AAR was identified by *in vivo* lectin staining, and expression of FAP, CD68, and hypoxic tissues were measured using immunohistochemistry. Spatial FAP was further interrogated by ^68^ Ga-FAPI-46 in mice by autoradiography and humans by PET. Additionally, human cardiac tissues from acute MI patients were examined for fibroblasts and inflammatory cells by expression of FAP, CD13, and α-smooth muscle actin.

**Results:**

FAP expression peaked three days post-MI/R predominantly within the AAR (p < 0.05 vs. d0). Consistent between murine models and human tissues, FAP^+^ myofibroblasts accumulated within the infarct scar and borderzone, occasionally extending into non-ischemic myocardium. CD68^+^ macrophages peaked similarly at three days post-MI/R (p < 0.05 vs. d0). FAP expression weakly correlated with CD68 but not with extent of ischemic or hypoxic territory post-MI/R. FAP imaging in mice and humans revealed aligned non-uniform ^68^ Ga-FAPI-46 uptake extending from the infarct scar into surviving myocardium after MI.

**Conclusions:**

Our findings demonstrate a distinct FAP expression pattern post-MI/R. The alignment of *ex vivo* ^68^ Ga-FAPI-46 signal with myofibroblasts in the AAR supports its identification of a unique substrate in myocardial injury complementing other non-invasive imaging measurements of perfusion, viability and fibrosis.

**Supplementary Information:**

The online version contains supplementary material available at 10.1007/s11307-025-01994-6.

## Introduction

Myocardial infarction (MI) is defined as myocardial cell death due to prolonged ischemia [1], and myocardial area at risk (AAR) as the heart tissue at risk of ischemic death beyond the territory supplied by the occluded artery. The acute response after MI includes inflammatory cell infiltration, fibroblast activation and formation of a mature scar within the infarcted myocardium [2, 3]. The irreversibly damaged portion of AAR varies widely wherein salvage of reversibly damaged myocardial tissue depends on factors including reperfusion timing, collateral flow and variations in cardiac wound healing and remodeling [4–7].

Quantifying the AAR and myocardial salvage together with non-invasive measurement of active wound healing *in vivo* could help evaluate novel treatments for acute MI, aiming to minimize ischemic damage [8].

Fibroblast activation protein alpha (FAP) is a cell surface glycoprotein on activated myofibroblasts after MI [9, 10]. Myofibroblasts are predominantly located at the interface between viable and infarcted myocardium and play a pivotal role in wound healing including production of extracellular matrix proteins and regulation of inflammation and angiogenesis through cytokine secretion post-MI [2, 11]. Recently, non-invasive imaging strategies using PET with radiolabeled FAP ligands have become available to characterize FAP expression in cardiovascular disease including acute ST-elevation myocardial infarction (STEMI) and severe aortic stenosis [12–14].

Preclinical and clinical findings from PET imaging early after acute MI indicate that FAP upregulation occurs within ischemic myocardium, but also extends beyond the persisting perfusion defect after reperfusion [12, 15–17]. Despite numerous studies evaluating FAP expression in the heart using PET, no study has yet analyzed the spatial pattern of FAP expression relative to scar and AAR after MI. Because spatial resolution of FAPI-PET is low, we aimed to analyze FAP-expression in a myocardial ischemia–reperfusion model in mice using immunohistochemistry, and complemented the results with autoradiography using ^68^ Ga-FAPI-46 distribution.

## Materials and Methods

Additional materials and methods are presented as Supplementary Online Material.

### Experimental Ischemia/Reperfusion in Mice

Animal studies were conducted in accordance with the principles and procedures outlined in the Guide for the Care and Use of Laboratory Animals and were approved by the local government (Niedersächsisches Landesamt für Verbraucherschutz und Lebensmittelsicherheit (LAVES), Lower Saxony, Germany). Ischemia/reperfusion (MI/R) was induced in male wildtype C57BL/6N mice (n = 19, purchased from Charles River) aged 10 weeks as described previously [18].

### *In vivo* Detection of Hypoxia and Perfused Vasculature

Harvesting of hearts in surviving animals was performed at three timepoints: immediately following ischemia (d0, *n* = 6), and at 3 days (d3, *n* = 4) or 7 days (d7, *n* = 6) after reperfusion. Briefly, Pimonidazole-HCl (Hypoxyprobe Inc., HP6-100) was injected i.v. for *in vivo* detection of hypoxia [19, 20]. Fluorescence-labeled lectin (Vector Laboratories, DL-1178–1) was injected i.v. for *in vivo* detection of perfused vasculature after reocclusion of the left anterior descending coronary artery (LAD) [21]. AAR was defined as ischemic area at risk identified by absence of lectin staining. Animals were euthanized by cervical dislocation under deep isoflurane anesthesia. The hearts were removed for histological analyses and frozen.

### Immunohistochemistry of Mouse and Human Myocardial Tissue

Frozen Sects. (4 µm) from mouse hearts were stained with antibodies against FAP, CD68 and pimonidazole and visualized by fluorescent labeling as described previously [10]. For expression analysis, a manual color threshold was applied to separate stained and background areas, and stained area fraction was determined as stained vs. total tissue area of interest for each image using NIS Elements 4.2 AR software (Nikon Instruments) [9, 22].

For human heart studies, FAP expression was evaluated in tissue from the left ventricular (LV) apex obtained from discarded tissue of patients receiving a LV assist device (LVAD) due to acute MI and severely reduced LV-function (*n* = 2). Biopsies obtained from non-failing donor hearts served as controls (*n* = 2). Briefly, Formalin fixed, paraffin embedded tissue samples were stained for FAP, CD13 or α-smooth muscle actin and visualized using chromogenic substrates DAB and Histogreen as described previously [22] and in supplementary methods B.4. CD13 was selected as a marker due to its established role in tissue remodeling processes, particularly its expression on activated fibroblasts, inflammatory cells, and endothelial cells during myocardial repair [22]. The study was approved by the ethics committee of Hannover Medical School and conforms to the ethical guidelines of the 1975 Declaration of Helsinki.

### ^68^ Ga-FAPI-46 Autoradiography Analysis in Mice

^68^ Ga-FAPI-46 was synthesized as previously described [23]. An additional group of mice was studied at 3 days after MI/R without LAD re-occlusion to assess FAP expression by *ex vivo* autoradiography using the PET radiotracer ^68^ Ga-FAPI-46 (*n* = 5) as described in supplementary methods B.5.

To compare the area of ^68^ Ga-FAPI-46 positive signal with histology defined fibrosis, we analyzed autoradiography and corresponding Masson-trichrome images using Fiji software[24]. Autoradiograms were colocalized with the histology images, and the areas of FAPI-positive signal, total tissue, and aniline blue-positive collagen were defined manually. The ratio of FAPI-positive signal to total tissue area and collagen to total tissue area were then calculated.

### ^68^ Ga-FAPI-46 Molecular Imaging in Patients with Acute MI

Patients provided written informed consent before imaging. Resting myocardial perfusion SPECT was performed at 3.7 ± 3.1 days after acute MI. Infarct size was measured with commercially available software for polar map generation (4DM, Invia, Ann Arbor, MI, USA) and compared against a local normal database. FAP-targeted PET (n = 7) was conducted at 4.9 ± 2.1 days after MI using the specific ligand ^68^ Ga-FAPI-46.

### Statistical Analyses

Data are presented as mean and standard error. We used Wilcoxon signed rank test for comparison of paired data, and Kruskal–Wallis one-way ANOVA with multiple comparisons for analysis of differences between more than two groups. We used linear regression analysis to identify measurements associated with FAP expression. To evaluate the relationship between FAP and CD68 expression, AAR or hypoxic area, we calculated the Spearman correlation coefficient. All analyses were performed with Prism 5.0 (GraphPad).

## Results

### FAP Expression and Inflammatory Cell Infiltration Increase within the AAR After MI/R

**Fig. 1:**
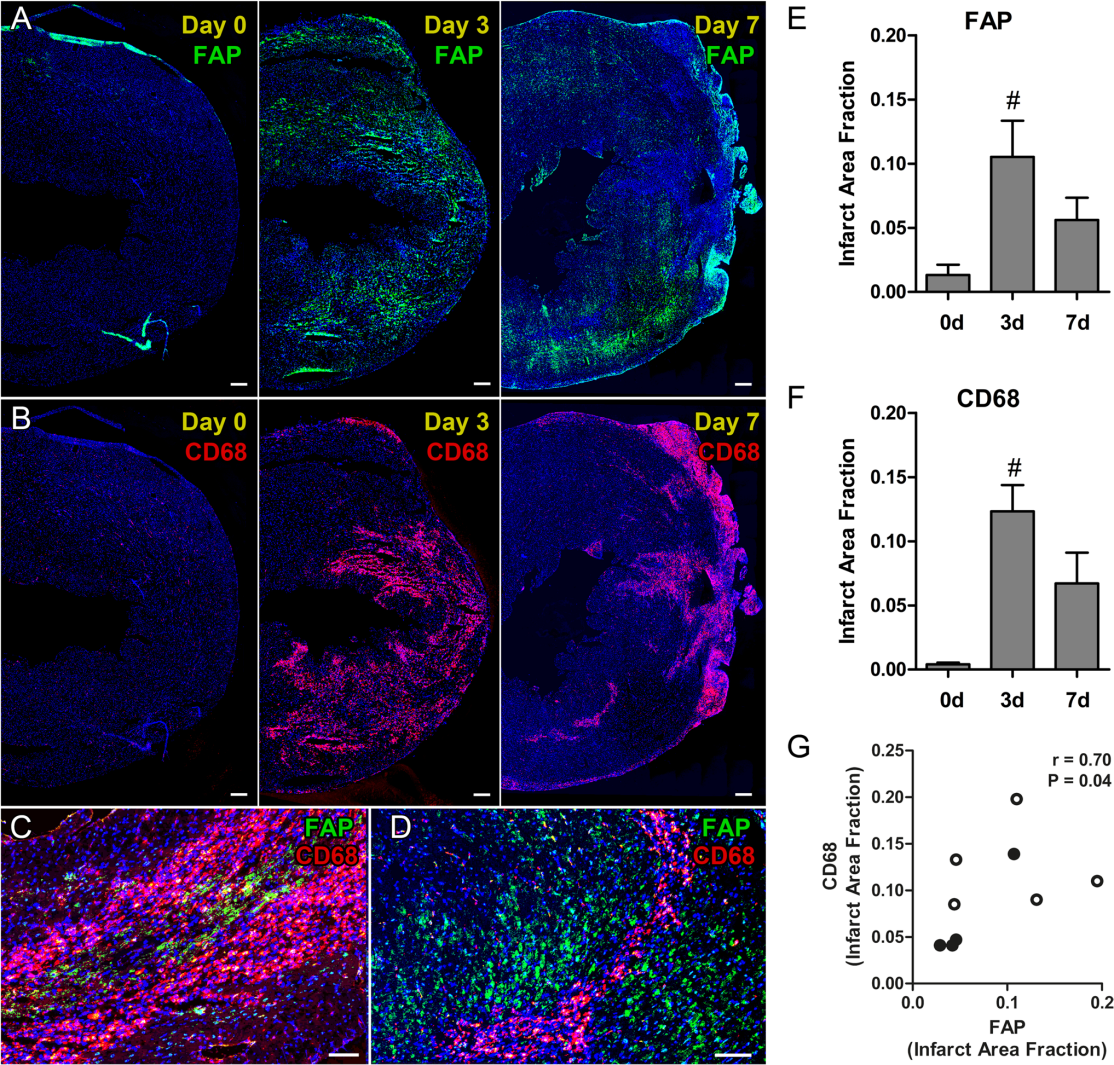
Time course of FAP and CD68 expression on day 0, 3 and 7 after MI/R. (**A**, **B**) Co-immunohistochemical analysis for FAP and CD68 in the same sections at day 0, 3, and 7 after MI/R. (**E**, **F**) FAP and CD68 expression peaked at day 3 in the infarcted myocardium. (**C**, **D**) FAP expressing myofibroblasts and CD68 expressing inflammatory cells were both located in close proximity within the infarcted myocardium, but were identified as distinct cell populations at 3 days (**C**) and 7 days after MI/R (**D**). (**G**) Weak correlation between FAP and CD68 at days 3 (white circles) and 7 (black circles). Bars indicate mean ± SE. MI/R day 0: *n* = 4; day 3: *n* = 5; day 7: *n* = 4, # = *p* < 0.05 vs. day 0. Scale bar: 200 µm (**A**, **B**) and 100 µm (**C**, **D**). FAP, green; CD68, red; Nuclei, blue

We performed multi-color fluorescence immunohistochemistry analyses in mouse hearts to characterize the accumulation of FAP^+^ myofibroblasts and CD68^+^ macrophages in the AAR. FAP^+^ and CD68^+^ cells were detected in the infarcted area at 3 and 7 days after MI/R, while both cell types were almost absent at day 0 after MI/R (Fig. 1A, B). Quantitatively, FAP and CD68 expression peaked at 3d and declined at 7d post-MI/R (Fig. 1E, F). There was a positive correlation between FAP^+^ and CD68^+^ expression at these time points (r = 0.70, *p* = 0.04; Fig. 1G). At 3d and 7d post-MI/R, FAP^+^ myofibroblasts and CD68^+^ macrophages were located in proximity within infarcted myocardium, but remained separate cell populations (Fig. 1C, D), underscoring that myofibroblast activation and monocyte recruitment are distinct mechanisms during the wound healing phase post-MI/R.

### Identification of AAR and Hypoxic Myocardium After MI/R in Mice

We verified the detection of AAR and hypoxia within LV myocardium at 0 and 3d post-MI/R (Suppl. Fig. 1). LAD-occlusion led to hypoxia in LV free wall myocardium located downstream the occluded LAD, while the septal wall remained normoxic (Suppl. Fig. 1A). Hypoxic areas were not different between d0 and d3 (d0: 2.6 ± 1.4 mm2; d3: 3.8 ± 1.1 mm2, *p* = 0.49). The AAR was identified by absence of lectin staining, indicating non-perfused vessels within myocardium (Suppl. Fig. 1B).

Hypoxia was detected throughout the AAR. Of note, the level of hypoxia was non-uniform within the AAR, and there was a narrow transition zone between absence of perfusion and presence of hypoxia at d0 (Suppl. Fig. 1C and 2). At d3 post-MI/R, the hypoxic area remained confined to the AAR, however there were more distinct patches without detectable hypoxia as compared to d0 (Suppl. Fig. 1D).

### FAP Expression Extends Into Surviving Myocardium and is not Correlated with AAR or Area of Hypoxic Myocardium 3 days After MI/R

**Fig. 2:**
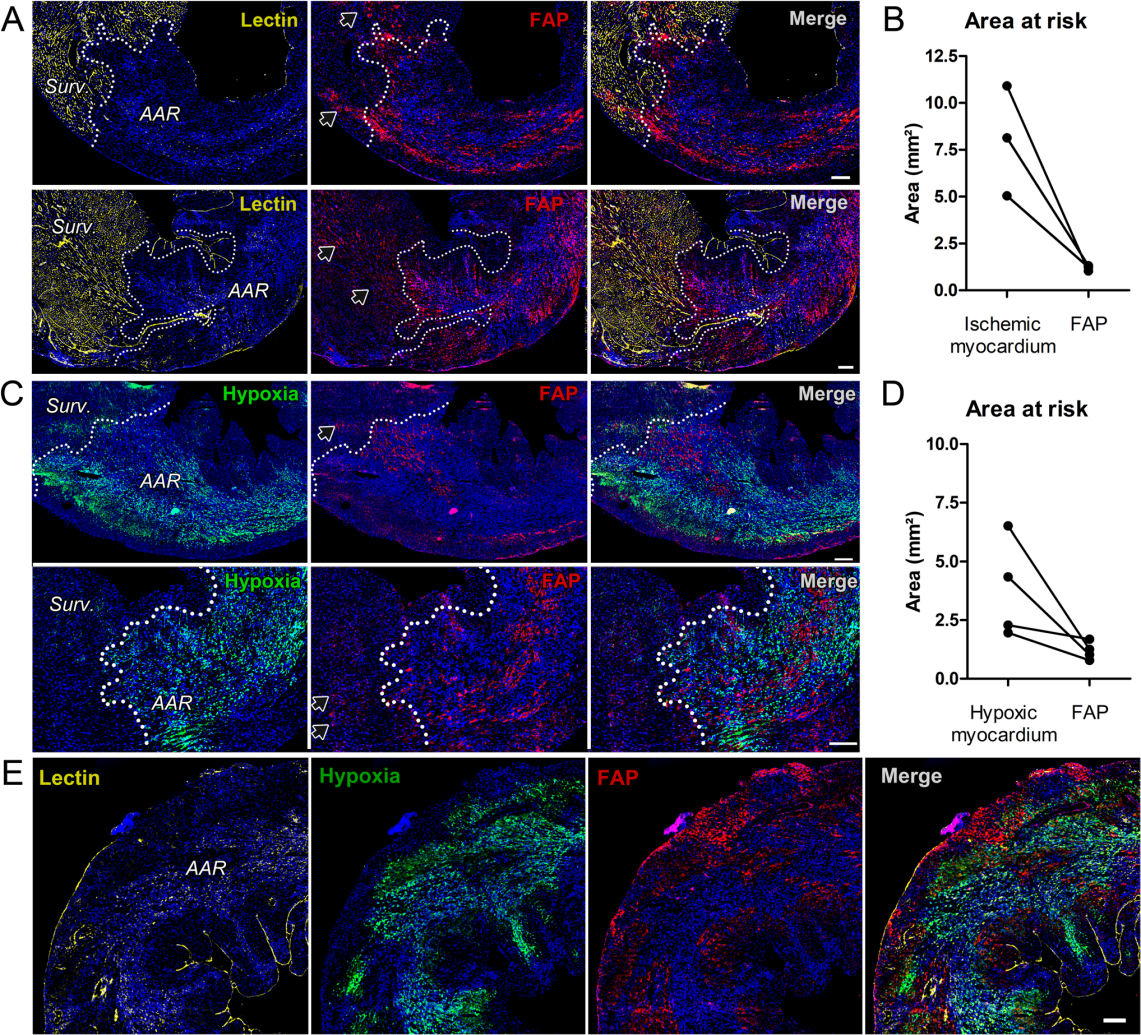
FAP expression in the AAR at day 3 after MI/R is not correlated with ischemic or hypoxic myocardium. (**A**, **C**) Co-immunohistochemistry for perfused myocardium, hypoxic myocardium, and FAP. The border between surviving (“surv.”) and ischemic myocardium (“AAR”) is delineated by dotted lines. FAP expression is predominantly located within the AAR in a non-uniform pattern, with little FAP expression detected in adjacent surviving myocardium (black arrows). (**B**, **D**) Quantitative analysis shows FAP expression is not correlated with ischemic (**B**) or hypoxic area (**D**). (**E**) Overview of ischemia, hypoxia and FAP-expression at 3 days after MI/R, demonstrating abundant FAP-expression within the AAR. Hypoxia (Pimonidazole), green; perfused vasculature (lectin), yellow; FAP, red; Nuclei, blue. (**B**) *n* = 3; (**D**) *n* = 4; Scale bars: 200 μm

Because FAP expression peaks at d3 post-MI/R, we performed quantitative co-localization analyses of FAP within AAR and hypoxic myocardium. The area of FAP expression was smaller than that of AAR (median, 8.1mm2 vs. 1.2mm2, n.s., Fig. 2B) and hypoxic myocardium (median, 3.3mm2 vs. 1.1mm2, n.s., Fig. 2D). FAP expression was non-uniformly distributed in distinct patches within the AAR, and correlated with neither AAR (r = −0.50, *p* = 1.0) nor hypoxic area (r = 0.40, *p* = 0.75). These results suggest that expression of FAP post-MI/R is predominantly related to myofibroblast activation within the infarcted myocardium but not directly related to the amount of ischemic tissue or tissue oxygen levels. Of note, while the majority of FAP expression was found within the AAR, FAP was also detected within surviving myocardium (Suppl. Fig. 3) and at the border to hypoxic areas and AAR (Fig. 2A, C).

### Uptake of ^68^ Ga-FAPI-46 is Located within Areas of Fibroblast and Inflammatory Cell Infiltration After MI/R, and Extends Beyond the Collagen Scar into the AAR

**Fig. 3:**
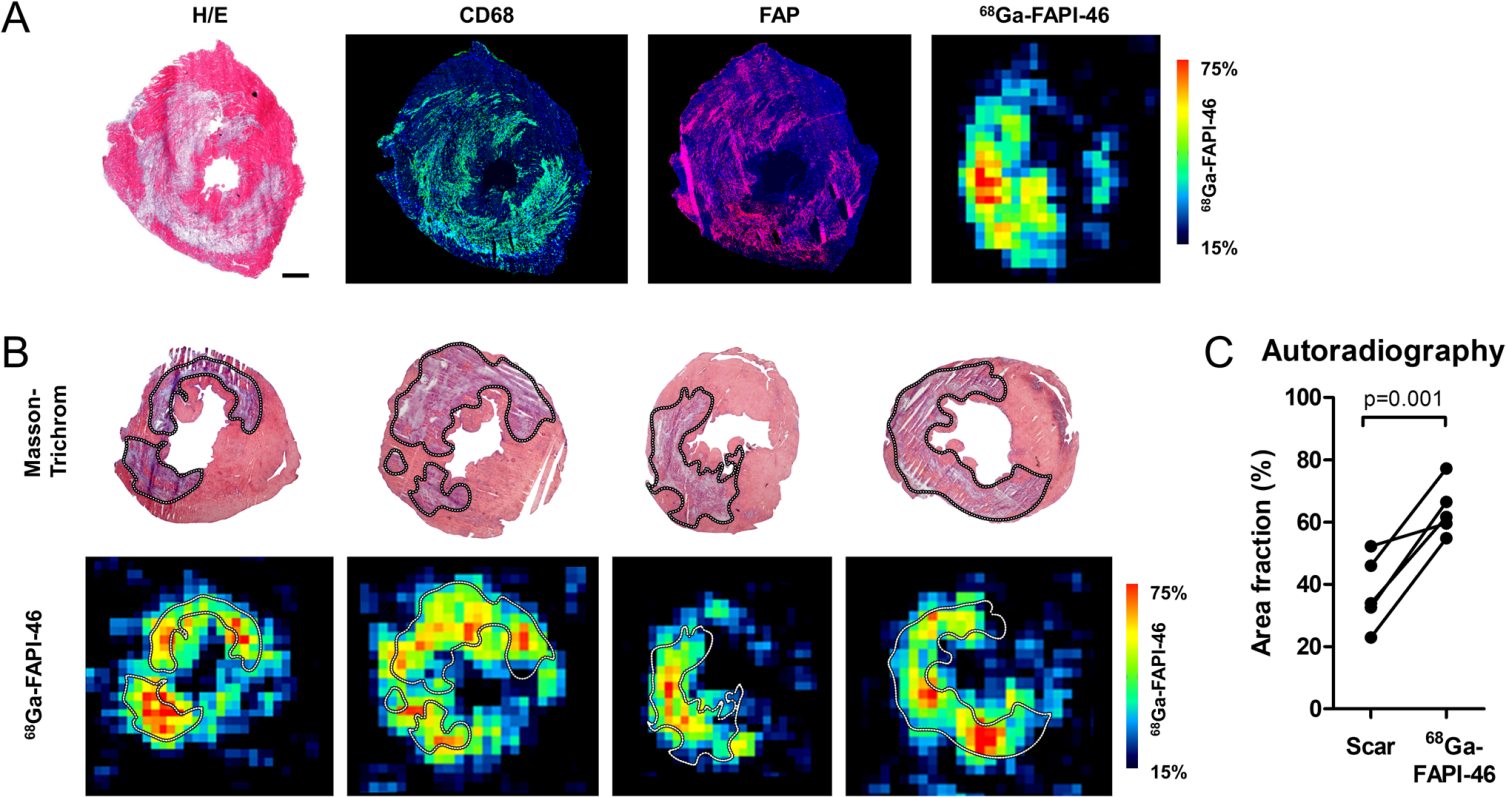
Myocardial ^68^ Ga-FAPI-46 autoradiography and FAP expression at 3 days after MI/R. (**A**) Representative example of FAP and CD68 expression together with ^68^ Ga-FAPI-46 autoradiography in nearby sections from the mid-portion of the infarcted heart. High uptake of ^68^ Ga-FAPI-46 tracer within the infarcted LV free wall as evidenced by hematoxylin–eosin (H/E) staining. Areas of FAP-expression and ^68^ Ga-FAPI-46 uptake are consistent across the sections. (**B**, **C**) ^68^ Ga-FAPI-46 uptake extends beyond the infarct scar. Masson trichrome staining delineates the collagen containing infarct scar (violet, dotted lines in upper panels), and ^68^ Ga-FAPI-46 autoradiography in the same sections shows uptake in the infarct area. ^68^ Ga-FAPI-46 uptake is non-uniformly distributed with spots of highest uptake in the infarct area but extends into the infarct borderzone and is also detected in remote myocardium. The area fraction of.^68^ Ga-FAPI-46 uptake in relation to the LV is higher than the area fraction of scar identified by Masson trichrome (*P* = 0.001) (**C**). CD68, green; FAP, red; Nuclei, blue. Scale bar: 200 μm. Autoradiography, *n* = 5

To verify uptake of ^68^ Ga-FAPI-46 in areas of FAP expression, we studied mice at d3 post-MI/R with immunohistochemistry and autoradiography in adjacent sections (Fig. 3). We found that uptake of ^68^ Ga-FAPI-46 matched expression pattern of FAP by immunohistochemistry, confirming the selectivity of the radiotracer for FAP. As expected, CD68^+^ macrophages were also present in the infarcted myocardium (Fig. 3A).

We further analyzed heart sections at d3 post-MI/R for collagen-expression within the infarct scar by Masson-trichrome staining and compared uptake of ^68^ Ga-FAPI-46 with collagen-distribution (Fig. 3B). We demonstrate that ^68^ Ga-FAPI-46 uptake extends beyond the infarct scar, again confirming that FAP is expressed throughout the AAR and infarct borderzone (Fig. 3C).

Of note, uptake of ^68^ Ga-FAPI-46 was non-uniformly distributed within the infarcted myocardium, with highest uptake in the myocardium with high collagen-expression (Fig. 3B).

### FAP is Upregulated After Acute MI in Humans in Perivascular and Reparative Fibrotic Tissue

**Fig. 4:**
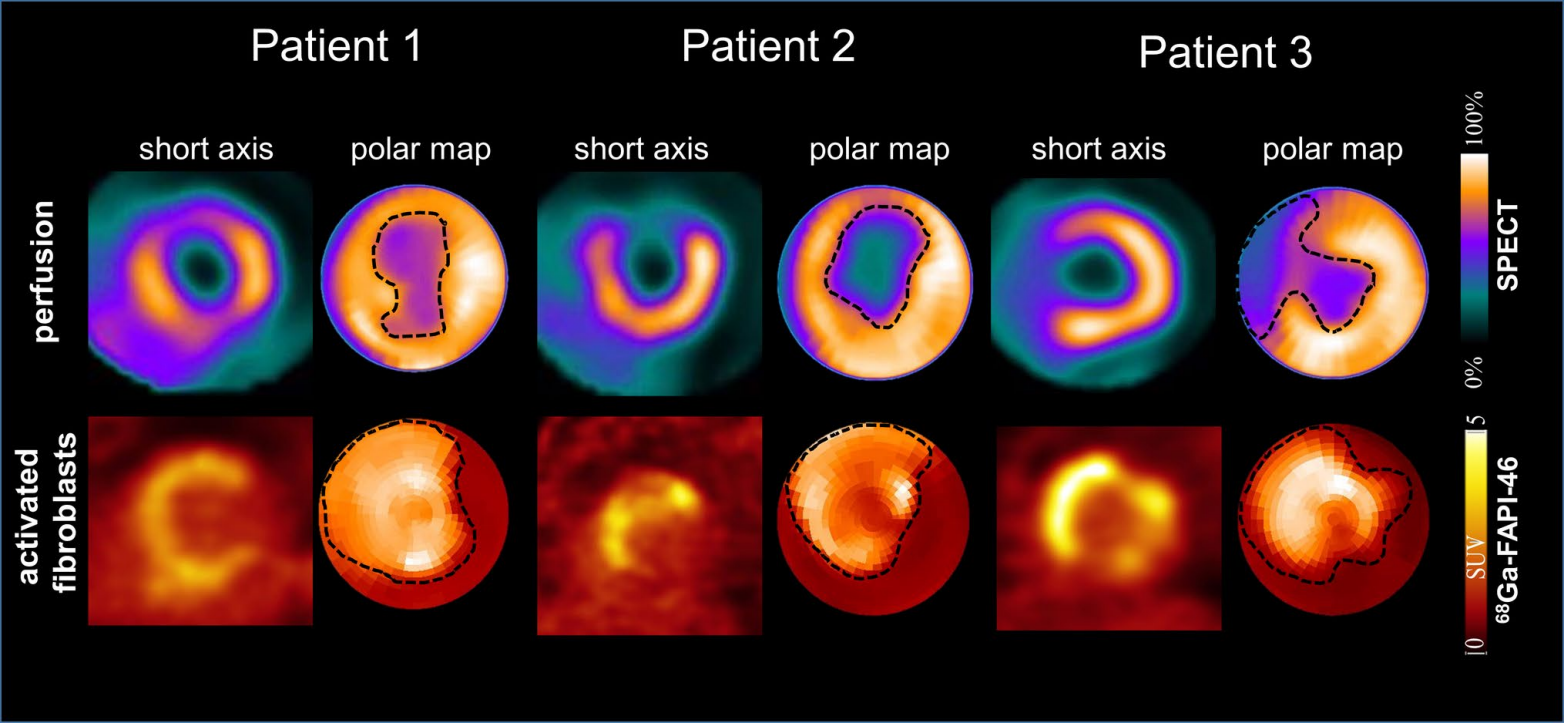
^68^ Ga-FAPI-46 PET signal exceeds the infarct region after anterior acute myocardial infarction. Depicted are: Myocardial perfusion images using 99mTc-tetrofosmin at rest (first row) and ^68^ Ga-FAPI PET (second row). Area of perfusion defect after revascularization and area of activated fibroblasts are indicated by dotted lines in 3 representative patient examples

To gain translational insight into distribution of FAP expression in human MI, we studied myocardial injury and FAP activation in patients with acute MI using myocardial perfusion SPECT and ^68^ Ga-FAPI-46 PET (Fig. 4). All patients showed markedly larger areas of elevated FAP signal compared to myocardial perfusion defect (62.9 ± 15.4% of LV vs. 23.9 ± 17.4% of LV; *p* = 0.003).

**Fig. 5:**
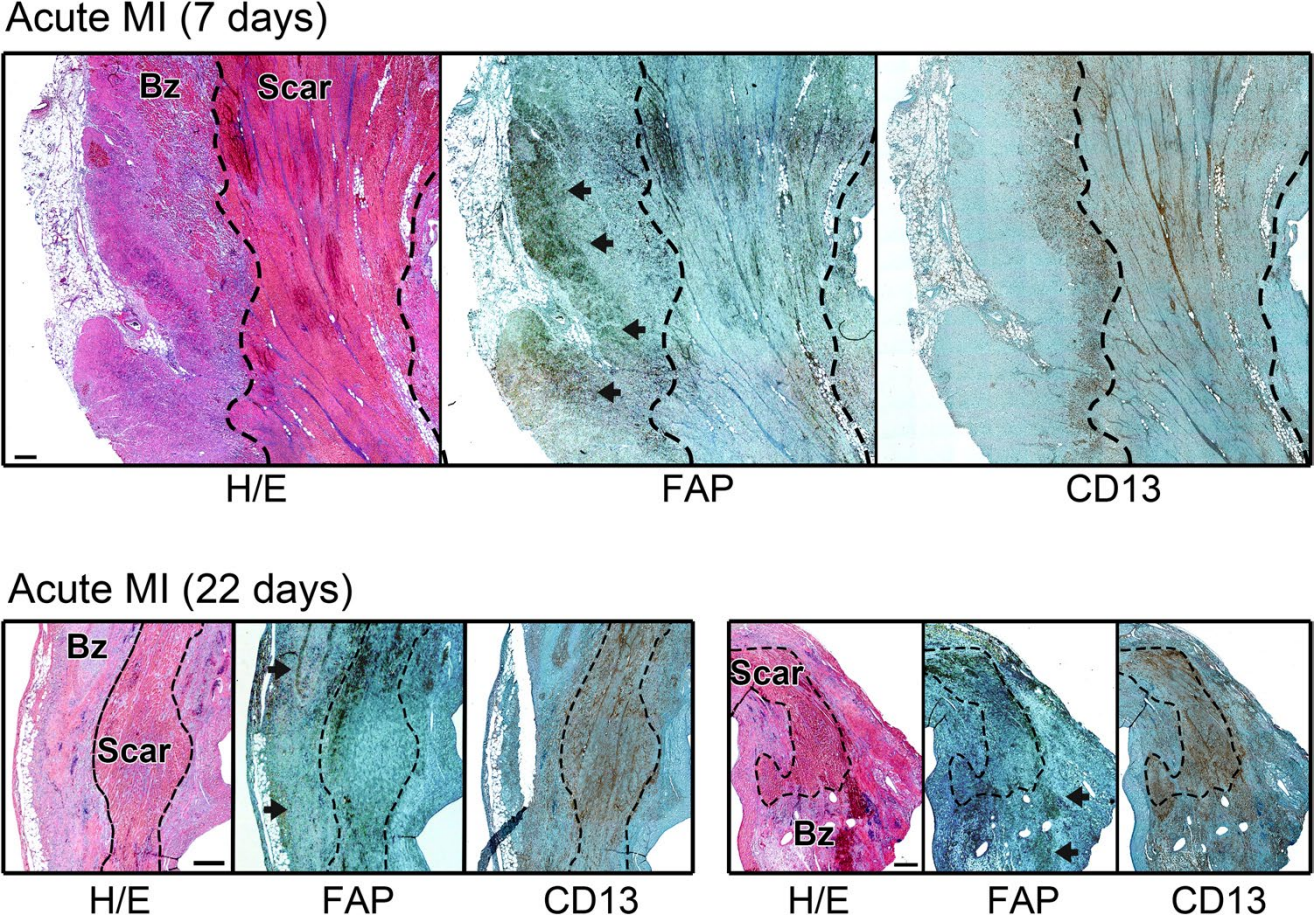
FAP expression exceeds the infarct region after acute MI in patients with reduced LV-function. Upper panels: 7 days post-MI, CD13 and FAP are expressed within the scar, borderzone and adjacent myocardial tissue. FAP is expressed adjacent to CD13-expressing cells in the border zone (arrowheads). Lower panels: 22 days post-MI, CD13 expression is found mostly within the scar, but extends into the granulation tissue of the border zone. FAP expression is non-homogenously distributed within the scar, and also found in clusters within the border zone and also around blood vessels (arrowheads). Scale bars: 500 μM. Bz, Borderzone

We then analyzed histological samples from individuals with severe heart failure due to acute MI by immunohistochemistry and healthy donor organs for transplantation (Fig. 5, Suppl. Fig. 4). Patient characteristics are detailed in Supplementary Data Table A.2. Infiltration with CD13^+^ interstitial fibrotic, inflammatory cells and pericytes surrounding blood vessels was evident in the infarcted LV myocardium (Suppl. Fig. 4), aligning with areas of active tissue remodeling. FAP-expressing myofibroblasts were located in the same areas, demonstrating that FAP is indeed expressed in areas of active tissue remodeling after acute MI. Of note, FAP was found not only in infarcted myocardium with reparative fibrosis, but also in surviving myocardium and around vascular structures in non-infarcted myocardium (Fig. 5, Suppl. Fig. 4).

## Discussion

Several studies reported increased uptake of FAP ligands in infarcted hearts post -MI, and correlation with clinical findings and magnetic resonance imaging (MRI) markers of myocardial damage, and even prediction of late LV remodeling [12, 13, 25]. Notably, FAP-expression was not only detected within the infarct scar, but also within the surviving myocardium as indicated by lack of direct alignment with late gadolinium enhancement (LGE) territory on MRI or the perfusion defect defined by SPECT [12, 13, 25]. This corresponds to histological findings in rat hearts demonstrating FAP expression extends beyond the infarct scar to the surviving myocardium near the necrotic area post-MI [10, 16]. To reconcile these findings, we characterized FAP-expressing cell distribution in necrotic and surviving myocardium post-reperfusion of experimental MI/R.

### Identification of the AAR after MI/R

We defined the AAR by absence of endothelial staining using *in vivo* lectin infusion to label capillaries and large vessels with high resolution [21]. Similar methods have been described including fluorescent microsphere injection and 2,3,5-triphenyltetrazolium (TTC) staining [6, 26]. Non-invasive radionuclide imaging of AAR faces spatial resolution limitations, which can be addressed in small animal models by *ex vivo* autoradiography. Differences in infarct and AAR have been characterized by multi-tracer autoradiography in a reperfused infarct rat model using thallium-201 and ^99m^Tc-Sestamibi radiotracers. Consistent with our study, autoradiography defined a remarkably larger AAR compared to the infarct scar [27]**.**

In humans, AAR can be estimated non-invasively by cardiac MRI, PET imaging or (99 m)Technetium (Tc)-Sestamibi SPECT [28–30]. However, these non-invasive methods are not performed routinely before coronary reperfusion in STEMI, making it challenging to identify initially perfused vs non-perfused myocardium retrospectively. In our study, *in vivo* lectin staining enabled precise AAR delineation post-MI/R using multi-color immunofluorescence and high -resolution microscopy surpassing the spatial resolution of current non-invasive techniques in humans.

Areas of hypoxia were indeed confined to the AAR, but the AAR was not exclusively hypoxic, especially at 3 days post-MI/R. Concordantly, hypoxic area was smaller than ischemic myocardium in individual animals. One explanation is that pimonidazole detects hypoxia at pO2 below 10 mmHg, whereas normal capillary bed pO2 is as low as 40 mmHg [31, 32]. An oxygen gradient within the infarct borderzone likely cannot detect intermediate hypoxia (10–40 mmHg) [33, 34], consistent with the narrow non-hypoxic transition zone at the border of ischemic and non-ischemic myocardium in our study. Moreover, early neovascularization of the reperfused myocardium might have improved oxygen supply to the scar despite LAD-occlusion [35].

However, we clearly identified the AAR by visualizing ischemic and non-ischemic myocardium. We further showed that hypoxic area is smaller than AAR, which can be reconciled given the known spatial and temporal heterogeneity of tissue oxygen in pathological conditions and neovascularization [32, 34].

### FAP Expression and Inflammatory Cell Infiltration Increase within the AAR After MI/R

Our study shows that FAP^+^ myofibroblasts and CD68^+^ macrophages infiltrate the infarcted heart with a peak at 3 days post-MI/R, often in close proximity but as distinct populations. This relationship may stem from myofibroblast activation via cytokines such as interleukin-1, −6, and TNF-α released by recruited macrophages and monocytes [3, 36]. MI/R leads to inflammatory cell invasion and myofibroblast activation, and requires tight temporospatial cell coordination for effective scar maturation [37, 38]. Ultimately, the healing infarct consists of cell debris and granulation tissue containing mostly inflammatory cells, myofibroblasts and endothelial cells [2, 38]. Supporting our findings, FAP^+^ fibroblasts and macrophages were also upregulated and co-localized in colorectal cancer tissues, where a synergistic cell–cell interaction network between these cell populations was identified [39].

FAP expression was non-uniformly distributed within the AAR, confined to areas with activated myofibroblasts in the scar and borderzone. In this respect, van den Borne et al. showed myofibroblasts occupy only ~ 5–10% of the infarct scar in mice [40]. Our results are also consistent with FAP expression in the infarcted area and borderzone after MI in rats [10, 16]. FAP^+^ cells were rarely detected in remote myocardium in both studies [10, 16].

Together, we show that FAP-expressing myofibroblasts are non-uniformly distributed within the AAR, with large FAP-negative regions.

### FAP Expression Extends Beyond the Infarct Scar After MI/R

Immunohistochemistry showed FAP^+^ myofibroblast area was smaller than the AAR or hypoxic myocardium, with no correlation between these areas and FAP expression. This reflects that FAP was expressed only in activated myofibroblasts, while other cells like inflammatory cells and cell debris remained FAP negative. Moreover, FAP expression was evident in the scar, in surviving ischemic myocardium and sometimes extended into surviving myocardium adjacent to the infarct borderzone, as evidenced by autoradiography. However, in contrast to immunohistochemical analysis the area of myocardium with ^68^ Ga-FAPI-46 uptake was larger than the scar area after MI/R. A similar uptake pattern was previously reported with ^68^ Ga-FAPI-04 [16].

Despite similar non-uniform distribution, extent of FAP expression detected by immunohistochemistry and ^68^ Ga-FAPI-46-PET imaging remains discrepant. This might be explained by several factors: First, the lower spatial resolution of PET imaging compared to immunohistochemistry may contribute to the apparent larger radiotracer uptake area, especially given that the infarcted ventricle wall thickness in mice tends toward the resolution limits. This issue is compounded by the high positron range of gallium-68 which may complicate definition of the boundary of the infarct and border zone. Notably, these complexities are mitigated in part in clinical application with larger structures. Second, the ^68^ Ga-FAPI-46 probe may be more sensitive to extracellular FAP, detecting soluble FAP in low concentrations that immunohistochemistry may miss, resulting in a broader detection range by PET imaging especially near infarct margins [41]. These differences highlight the need for further studies to clarify the mechanisms underlying the varying detection of FAP across these methodologies.

Increased FAP expression within the AAR, borderzone and adjacent non-ischemic surviving myocardium can be explained by cytokine release post-MI/R. The borderzone, adjacent to the infarct, involves intact tissues with ischemia-specific transcriptomic changes [42]. This area is clinically relevant, since it can extend into non-injured myocardium during post-MI remodeling [4, 42, 43]. Diekmann et al. found FAP uptake correlated with perfusion defect size by SPECT and LGE by MRI, but the area of FAP uptake was about twice as large, indicating that ^68^ Ga-FAPI-46 uptake provides a measurement distinct from perfusion defect or tissue fibrosis. Moreover, FAP expression and LGE signals often mismatched in segmental infarct analysis, supporting a distinction between FAP^+^ cells and tissue fibrosis [13].

Thus, FAP may serve as a potential marker for myocardial injury extent and healing, complementing MRI, PET, and SPECT in humans, particularly targeting transient activated myofibroblasts in the early stage of disease [13, 16, 25, 44].

### FAP is Upregulated After Acute MI in Humans in Perivascular and Reparative Fibrotic Tissue

Lastly, we demonstrate that FAP^+^ fibroblasts are present at low frequency within surviving areas near the infarct scar in humans with acute MI, consistent with previous findings [22]. The expression of CD13 demonstrated the spatial localization of FAP expressing cells in relation to fibroblast activation, inflammatory response and angiogenesis within the infarct and border zones during myocardial healing post-MI/R.

Recent studies highlighted the therapeutic potential of FAP + myofibroblasts, wherein cardiac fibrosis after angiotensin II and phenylephrine infusion was nearly eliminated by ablating FAP^+^ cells with antigen-specific CD8^+^ T cells [45].

Using clinical imaging we demonstrate that the myocardial area showing FAP upregulation exceeded the infarct region in patients shortly after acute MI and standard reperfusion therapy consistent with prior reports [12, 13]. The region of fibroblast activation on ^68^ Ga-FAPI PET extended beyond the perfusion defect visible with conventional SPECT. This finding was supported by cellular FAP expression outside the infarct area using immunohistochemistry in acute MI patients. This suggests that FAP upregulation plays a role in both replacement fibrosis in the injured area and reactive fibrosis in non-infarcted myocardium. A first report has shown a prognostic role of PET-derived fibroblast activation extent for later LV dysfunction [13].

### Study Limitations

Our study was performed in mice, which exhibit variable wound healing responses post-MI depending on strain and gender [7, 40]. However, our results are consistent with findings in rats [10], and we showed that also in humans FAP^+^ cells were occasionally found outside the infarcted area in acute MI. We observed variability of ischemic areas between animals, and statistical significance was not reached likely due to due to the small sample size. Nevertheless, we demonstrated the unique pattern of FAP expression in ischemic and non-ischemic myocardium extending beyond the necrotic scar. Another limitation of our study is the lack of long-term timepoints for evaluation of FAP expression. Still, our study found maximum FAP expression at 3 days post-MI/R, and we previously demonstrated that FAP activity decreases afterwards [10]. Therefore, FAP distribution within the AAR would likely be smaller than observed at 3 days post -MI/R at later timepoints.

## Conclusion

Our study is the first to demonstrate a distinct pattern of FAP expression primarily within and to a lesser extent beyond the AAR early post-MI/R. This explains findings from ^68^ Ga-FAPI-46 radioimaging, which detected non-uniform FAP expression within and beyond the infarct, highlighting the role of non-invasive FAP imaging in assessing myofibroblast activity complementary to other non-invasive imaging techniques such as MRI, FDG-PET, and SPECT. Further studies are warranted to demonstrate how non-invasive FAP imaging can support therapies aimed at reducing infarct size, fibrosis, and ischemic heart failure after MI.

## Supplementary Information

Below is the link to the electronic supplementary material.

Supplementary file1 (DOCX 32102 KB)

## Data Availability

The datasets generated during and/or analyzed during the current study are available from the corresponding author upon reasonable request.

## Abbreviations

LV: Left ventricular
FAP: Fibroblast Activation Protein alpha
AAR: Area at risk
MI: Myocardial infarction
MI/R: Myocardial ischemia/reperfusion
LAD: Left anterior descending coronary artery
