# Supplementary material for: Spatial FAP Expression as Detected by 68 Ga-FAPI-46 Identifies Myofibroblasts Beyond the Infarct Scar After Reperfusion
Source: Mol Imaging Biol. 2025 Mar 3;27(2):173–83. doi: 10.1007/s11307-025-01994-6 (PMC12062164; doi:10.1007/s11307-025-01994-6)
Supplement: Supplementary file 1 — Supplementary file1 (DOCX 32102 KB) [file 11307_2025_1994_MOESM1_ESM.docx]

# Appendix A - Tables

## Table A.1. Antibodies used for immunohistochemistry

| **Protein/Target** | **Antibody** | **Manufacturer** | **Labeling** | **Chromogen, Fluorochome(s) ^1^** |
| --- | --- | --- | --- | --- |
| Nuclear DNA | DAPI | Invitrogen | - | DAPI |
| FAP | Rabbit polyclonal | Abcam | Fluorescence, unconjugated | DL488 Cy3 |
| CD68 | Rat monoclonal | Invitrogen | Fluorescence, unconjugated | DL488  Cy3 |
| pimonidazole HCl | Rat monoclonal | Hypoxyprobe, Inc | Fluorescence, conjugated | FITC |
| Lycopersicon Esculentum (Tomato) Lectin | - | Vector | Fluorescence, conjugated | DL594, DL649 |
| FAP | Sheep polyclonal | R&D | Chromogenic | DAB |
| CD13 | Rabbit monoclonal | Abcam | Chromogenic | DAB |
| α-Smooth muscle actin | Mouse monoclonal | Sigma aldrich | Chromogenic | HG |

^1^ Abbreviations: FITC, Fluorescein Isothiocyanate; Cy3, Cyanine 3 dye; DL488, DyLight 488; DL594, DyLight 594; DL649, DyLight 649; DAB, 3,3'-Diaminobenzidine; HG, HistoGreen

## Table A.2. Characteristics of patients with acute MI for histological analysis

| Gender | Age | Days since MI | LV  Ejection fraction | Infarct related arteries |
| --- | --- | --- | --- | --- |
| M | 18 | 22 | <10% | main stem, LAD and LCX occlusion |
| M | 53 | 7 | 13% | LAD occlusion |
|  | | | | |

LAD: left anterior descending coronary artery. LCX: left circumflex artery

# Appendix B - Supplementary Materials and Methods

## B.1. Experimental ischemia/reperfusion in mice

Under 2% isoflurane anesthesia (induction with 4% isoflurane), the thorax was opened and the proximal left anterior descending coronary artery was occluded using a 7–0 suture around a piece of PE10 tubing. Sixty minutes after the coronary occlusion the suture was released to allow reperfusion, and the thorax closed with the open suture left in place. Animals were kept warm with a heating pad. Depth of anesthesia was tested using the pedal withdrawal reflex. Analgesia was ensured by an injection of butorphanol (2mg/kg BW s.c.) prior to surgery, and maintained using tramadol (1mg/ml in drinking water) for five days. Before and after surgery, animals were housed in groups and monitored daily for activity and signs of pain.

## B.2. In vivo detection of hypoxia and perfused vasculature

Harvesting of hearts in surviving animals was performed at three timepoints: immediately following ischemia (d0, n=6), and at 3 days (d3, n=4) or 7 days (d7, n=6) after reperfusion. For the latter two timepoints, under isoflurane anesthesia, the thorax was re-opened and the tail-vein was cannulated with a 29G catheter. For *in vivo* detection of hypoxia, 1.5mg of pimonidazole-HCl (Hypoxyprobe Inc., HP6‑100) was injected slowly i.v. and allowed to circulate and distribute to tissues for 15 minutes [[1-2](#_ENREF_1)]. Under hypoxic conditions, pimonidazole is stabilized and binds to intracellular proteins where it can be detected by immunohistology, while unbound pimonidazole is metabolized [[3](#_ENREF_3)]. Subsequently, the proximal left anterior descending coronary artery (LAD) was re-occluded for sixty minutes using the existing suture to induce myocardial ischemia. For *in vivo* detection of perfused vasculature, 50µg of fluorescence-labeled lectin (Vector Laboratories, DL‑1178‑1) was injected via tail vein and allowed to circulate for at least 5 minutes after 60min of reocclusion [[4](#_ENREF_4)]. For the MI/R day 0 group, all procedures were performed in one session without reperfusion. Animals were euthanized by cervical dislocation under deep isoflurane anesthesia. The hearts were removed for histological analyses and frozen.

## B.3. Immunohistochemistry of mouse myocardial tissue

At sacrifice, mouse hearts were washed in saline, embedded in OCT compound (Tissue-Tek) and frozen for subsequent analyses. Short-axis sections of 4µm thickness were fixed with ice-cold acetone. For immunofluorescence stainings, a blocking-step using 10% donkey serum in PBS was followed by sequential incubation with primary antibodies against FAP, CD68 or pimonidazole. FAP and CD68 antibodies were then detected by fluorescent secondary antibodies (Jackson ImmunoResearch). Nuclear DNA was labeled using DAPI (Invitrogen). Antibodies and fluorochromes are listed in Supplementary Data Table A.1.

Fluorescence images of whole transversal myocardial sections were taken using a Nikon NiE microscope with motorized stage at 20x magnification, and single images were stitched to obtain high-resolution representations of whole sections. Quantification of marker expression was performed for FAP and CD68 within infarcted myocardium of the left ventricle at d0 (n=4), d3 (n=5) and d7 (n=4) after MI/R. Quantified areas ranged from 3.6 to 11.2 mm². Quantitative analysis of hypoxic (n=3) and non-perfused area (n=4) was performed at day 3 after MI/R. Image processing with Photoshop (Adobe) included changes in brightness, contrast and tonal range, and was applied equally across the entire image.

## B.4. Immunohistochemistry of human myocardial tissue

For human heart studies, FAP expression was evaluated in paraffin-embedded formalin-fixed tissue from the left ventricular apex obtained from discarded tissue of male patients (n=2) receiving a left ventricular assist device (LVAD) due to acute MI (see also Table A.2). Biopsies obtained from non-failing donor hearts served as controls (n=2), as described previously [[5](#_ENREF_5)]. Human breast cancer tissue served as positive control for detection of FAP expression.

Formalin fixed, paraffin embedded tissues were sectioned at 4 µm, and heat induced antigen retrieval was performed using Histosafe Enhancer (Linaris, Germany). After blocking with 10% horse serum, sections were incubated with primary antibodies for FAP, CD13 or α-smooth muscle actin, followed by biotinylated secondary antibodies (Vector, Burlingame CA, USA), avidin-biotin-complex (Vector) and 3,3'-Diaminobenzidine (DAB, Abcam, Cambridge, UK) or HistoGreen (Linaris, Germany) as chromogenic substrate [[5-6](#_ENREF_5)]. Antibodies and chromogens are listed in Supplementary Data Table A.1. Sections were counterstained with Hematoxylin to identify nuclei.

Brightfield images were taken using a Nikon NiE microscope with motorized stage at 10x magnification, and single images were stitched to obtain high-resolution representations of whole sections. Image processing with Photoshop (Adobe) included changes in brightness, contrast and tonal range, and was applied equally across the entire image.

## B.5. ^68^Ga-FAPI-46 autoradiography analysis in mice

LAD was not re-occluded to allow tracer accumulation within the whole myocardium. Animals were euthanized 60 min after i.v. administration of 12.2±0.1 MBq of ^68^Ga-FAPI-46 under anesthesia. The hearts were excised, rinsed in cold PBS, frozen and cut into 10-µm short axis sections that were exposed to a phosphor imaging screen for 60 min and digitized using a high resolution radioluminography laser scanner (Raytest, CR 35 Bio, Straubenhardt, Germany). The resulting data was analysed using Pmod Version 3.7 (PMOD Technologies, Switzerland). Hearts used for autoradiography were subsequently stained with hematoxylin-eosin and Masson trichrome for gross histological evaluation and alignment to autoradiograms.

## B.6. ^68^Ga-FAPI-46 molecular imaging in patients with acute MI

FAP-targeted PET (n=7) was conducted at 4.9±2.1 days after MI using the specific ligand ^68^Ga-FAPI-46, synthesized in-house according to good manufacturing practice and used clinically per §13.2b of the German Pharmaceuticals Act to determine myocardial injury. Static PET images were acquired for 20 minutes using a Biograph mCT 128 PET/CT system (Siemens, Knoxville, TN, USA), starting 60 minutes after intravenous injection of 135.4±12.2 MBq of ^68^Ga-FAPI-46. Low-dose computed tomography (CT) scans were used for attenuation correction. Images were iteratively reconstructed using time-of-flight and point-spread function information (True X, Siemens). Area of fibroblast activation was measured with commercially available software (syngo.via; V50B, Siemens Healthcare).

## B.7. References

1. Sinusas AJ (1999) The potential for myocardial imaging with hypoxia markers. Semin Nucl Med 29:330-338.

2. Zampino M, Yuzhakova M, Hansen J, et al. (2006) Sex-related dimorphic response of HIF-1 alpha expression in myocardial ischemia. Am J Physiol Heart Circ Physiol 291:H957-964.

3. Williams MV, Denekamp J, Minchinton AI, Stratford MR (1982) In vivo assessment of basic 2-nitroimidazole radiosensitizers. Br J Cancer 46:127-137.

4. Robertson RT, Levine ST, Haynes SM, et al. (2015) Use of labeled tomato lectin for imaging vasculature structures. Histochem Cell Biol 143:225-234.

5. Tillmanns J, Schneider M, Fraccarollo D, et al. (2015) PET imaging of cardiac wound healing using a novel [68Ga]-labeled NGR probe in rat myocardial infarction. Mol Imaging Biol 17:76-86.

6. Tillmanns J, Hoffmann D, Habbaba Y, et al. (2015) Fibroblast activation protein alpha expression identifies activated fibroblasts after myocardial infarction. J Mol Cell Cardiol 87:194-203.

# Appendix C – Supplementary Figures

## C.1. Supplementary Figure 1

##
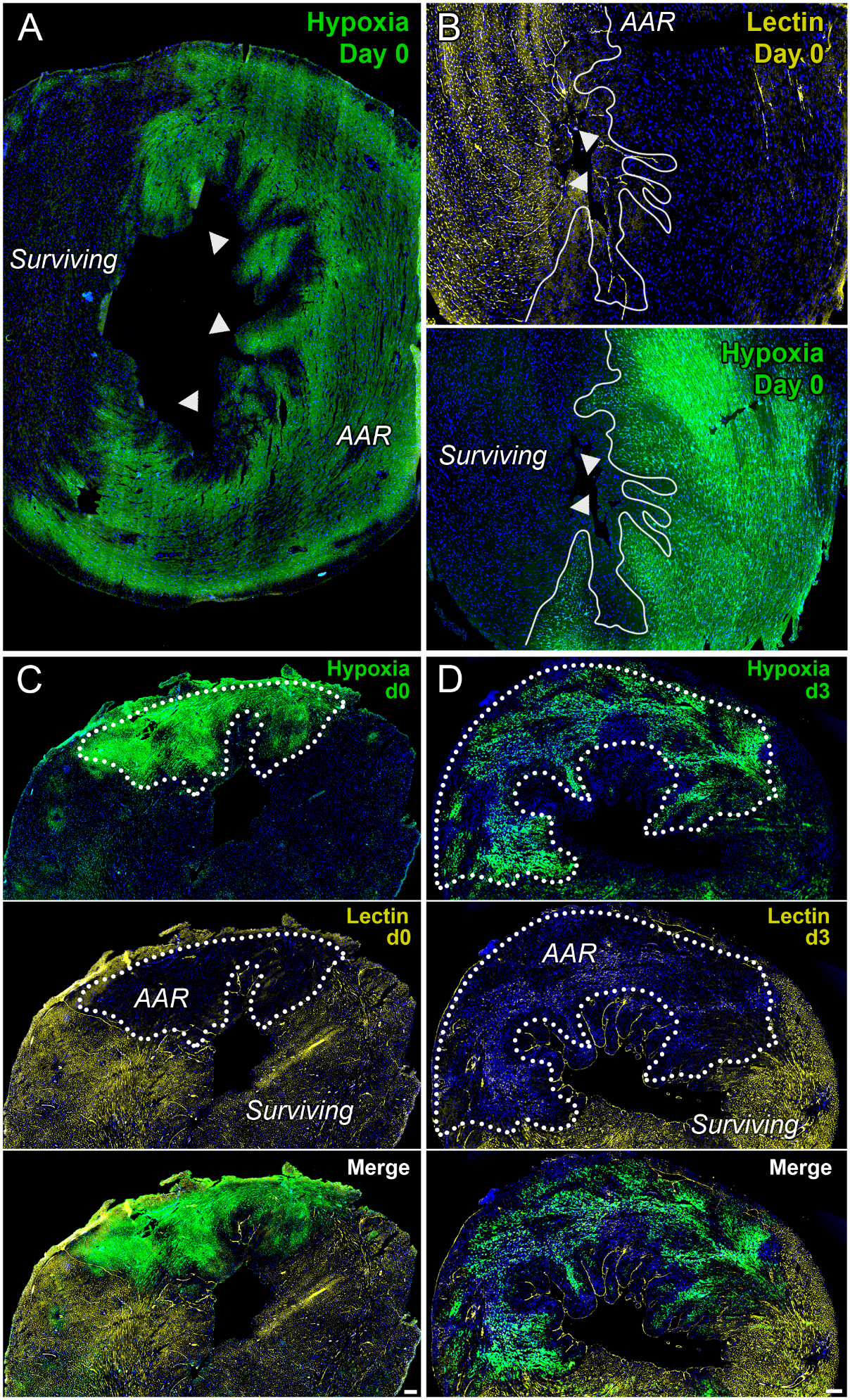


Supplementary Figure 1: Ischemia and hypoxia induced by experimental MI/R at day 0 and 3. (A) Sixty minutes after LAD occlusion, large parts of the LV free wall were hypoxic (green), while the LV septum was non-hypoxic. (B) Hypoxia was confined to the non-perfused area at risk (AAR). Transition zones between perfused ("surviving," yellow) vs. ischemic areas ("AAR") and non-hypoxic (“surviving”) vs. hypoxic areas (“AAR”, green) after MI/R (solid white line) were congruent. Surviving myocardium shows perfusion during LAD occlusion (B, upper panel). Myocardium surrounding the LV cavum remained non-hypoxic due to persistence of perfusion in the endocardium (A and B, arrowheads).

(C, D) Hypoxic myocardium (green) and non-perfused myocardium (AAR) at 0 days (left panels, C) and 3 days (right panels, D) after MI/R. Hypoxia developed within the non-perfused AAR of the LV free wall (dotted lines). Not all ischemic areas developed detectable hypoxia especially at day 3 after MI/R, likely due to neovascularization within the infarcted area.

*H*ypoxia (Pimonidazole), green; perfused vasculature (lectin), yellow; Nuclei, blue. Scale bars: 200μm.

## C.2. Supplementary Figure 2


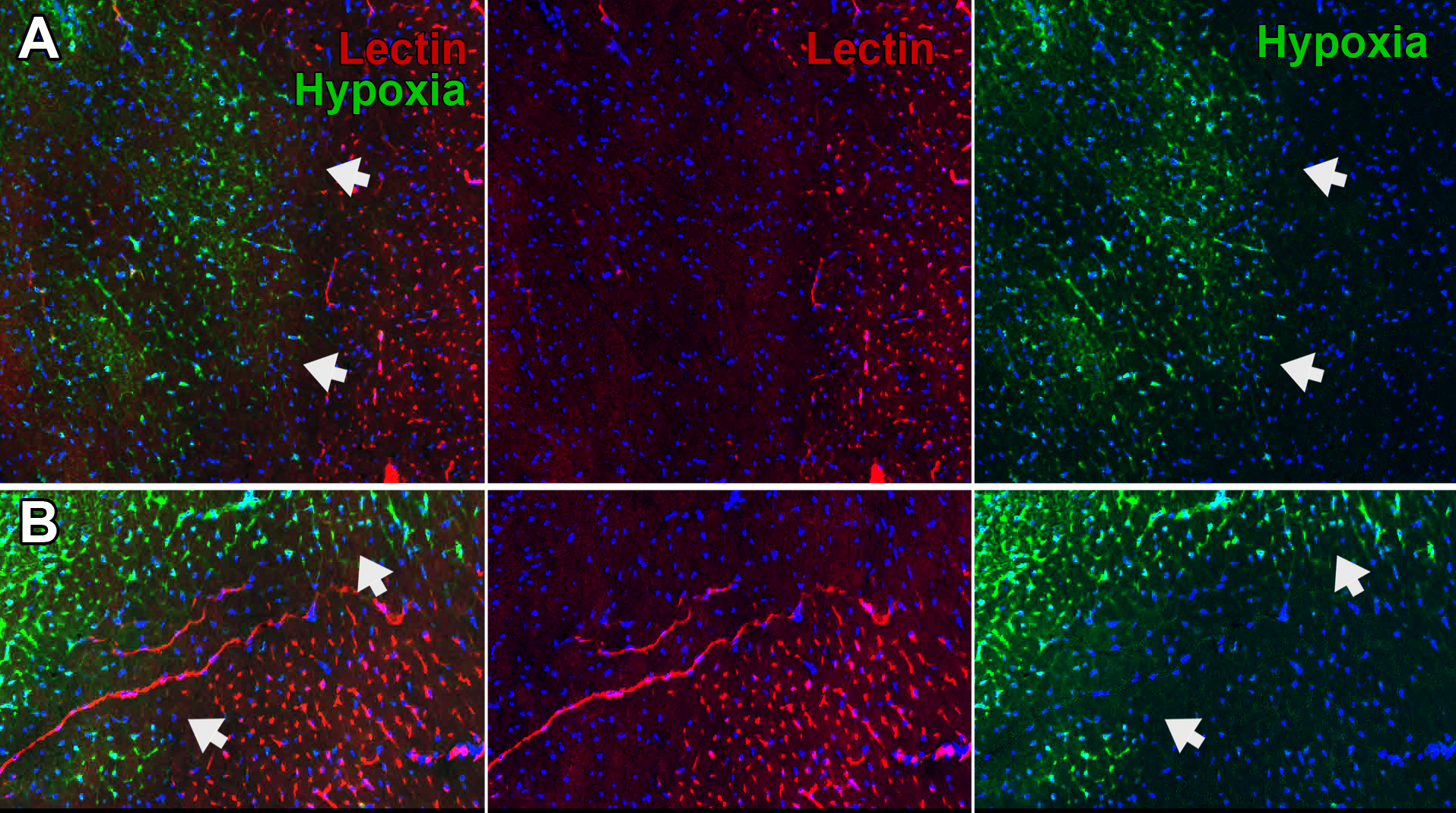


Supplementary Figure 2: A, B: Higher magnification image details of the transition zone between perfused and ischemic myocardium, demonstrating a narrow gap (white arrows) between perfused vasculature (red) and induction of hypoxia (green), likely reflecting an oxygen gradient between perfused and non-perfused myocardium after MI/R.

*In vivo* hypoxia (Pimonidazole), green; *in vivo* perfused vasculature (lectin), red; Nuclei, blue. Image processing included changes in brightness, contrast and tonal range, and was applied equally across the entire image.

## C.3. Supplementary Figure 3


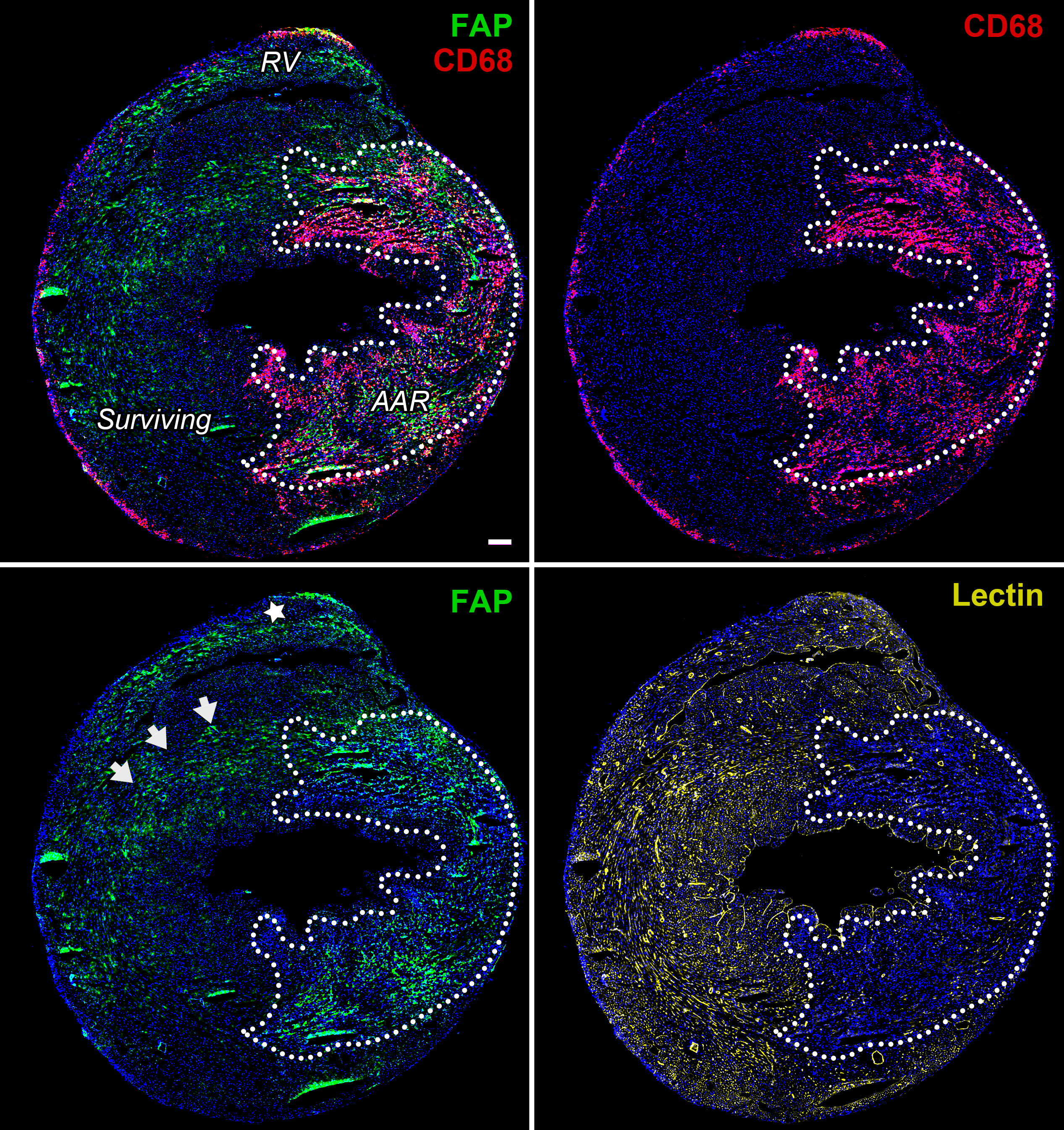


Supplementary Figure 3: FAP is expressed beyond the ischemic myocardium at day 3 after MI/R. Multicolor immunofluorescence image of the same section stained simultaneously with FAP (red), CD68 (green), Lectin (yellow). Upper left panel: Overlay of FAP, CD68 and lectin staining, showing AAR, surviving myocardium and right ventricle (RV). The AAR is clearly identified as non-perfused myocardium (lower right panel). CD68^+^ macrophages are predominantly located within the AAR (upper right panel). FAP is similarly expressed in patches within the AAR, but extends into the surviving myocardium (arrows) and is also expressed in parts of the RV (asterisk) (lower left panel).

FAP, red; CD68, green; *in vivo* perfused vasculature (lectin), yellow; Nuclei: blue. Scale bar: 200µm. Image processing included changes in brightness, contrast and tonal range, and was applied equally across the entire image.

## C.4. Supplementary Figure 4


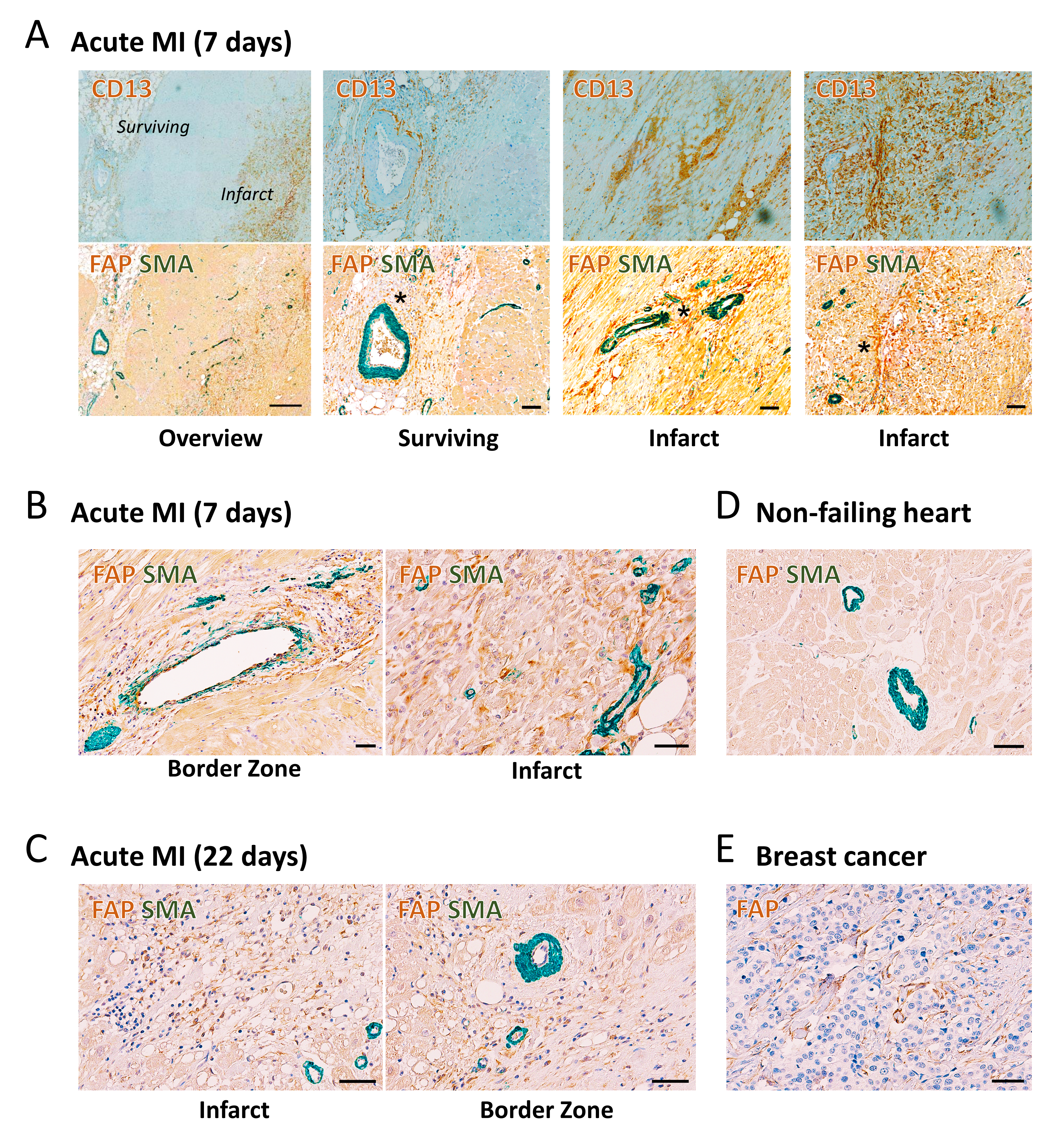


Supplementary Figure 4: Characterization of FAP expression in hearts of two patients with acute MI and severely reduced LV-function. A: Areas of myofibroblast and inflammatory cell activation were characterized by expression of CD13 (upper panels). FAP was expressed in myofibroblasts surrounding vessels and dispersed in reparative fibrotic tissue (black stars, lower panels), and at times around large vessels in surviving myocardium.

B, C: Higher magnification images of FAP in human hearts. FAP is expressed in areas reparative fibrosis and around large blood vessels in the infarcted area and border zone at 7 days (B) and 22 days after MI (C). D: FAP is not expressed in myocardial tissue of patients without overt heart disease. E: Human breast cancer tissue served as positive control for FAP-expression.

A: Upper panels: CD13, brown. Lower panels: FAP, brown; α-smooth muscle actin, green. B-D: FAP, brown; α-smooth muscle actin, green. E: FAP, brown; A-E: Nuclei, blue; Scale bars: 100μM (A), 50μM (B-E). Image processing included changes in brightness, contrast and tonal range, and was applied equally across the entire image.
